# Evaluation of Hepatitis B Vaccine Immunogenicity in Low-Birth-Weight Infants After Complete Immunization: The Impact of Postnatal Catch-Up Growth and Maternal–Neonatal Characteristics

**DOI:** 10.3390/vaccines14070566

**Published:** 2026-06-27

**Authors:** Lu Shen, Wanqin Tang, Yan Xie, Ran Hu, Jintao Wang, Yunke Qian, Huaxian Liu, Yang Yu, Zhongkui Zhu

**Affiliations:** 1 Department of Expanded Programme on Immunization, Taizhou City Center for Disease Control and Prevention, Taizhou 225300, China; hlxysl@163.com (L.S.);; 2 Department of Expanded Programme on Immunization, Jiangsu Provincial Center for Disease Control and Prevention, Nanjing 210009, China

**Keywords:** low birthweight infants, hepatitis B vaccine, immunogenicity, seroprotection, postnatal growth

## Abstract

**Background**: Low-birth-weight (LBW, <2500 g) infants are at increased risk of suboptimal hepatitis B vaccine responses; yet, data on their immunogenicity patterns and modifiable determinants remain limited. This study aimed to assess hepatitis B vaccine immunogenicity in LBW infants and to examine whether postnatal catch-up growth and maternal–neonatal characteristics are independently associated with antibody levels. **Methods:** We enrolled 511 LBW infants who completed the recommended 3-dose hepatitis B vaccination series at 0, 1, and 6 months. Blood samples were collected 4–6 weeks after completion of the full vaccination series. Geometric mean concentration (GMC) and seroprotection rate (SPR, anti-HBs ≥ 10 mIU/mL) were evaluated. Catch-up growth was quantified as the change in weight-for-age Z-score between 6 and 8 months of age (ΔWAZ). Multivariable linear regression was used to identify independent predictors of log-transformed antibody titers, adjusting for gestational age, maternal hepatitis B surface antigen (HBsAg) positivity, maternal body mass index (BMI), maternal fasting glucose, maternal thyroid disease, infant hemoglobin at 6 months, and ΔWAZ. **Results:** The overall SPR was 99.41% (508/511), with a GMC of 1045.37 mIU/mL (95% CI: 916.24–1192.70). SPR remained consistently high across all subgroups. In multivariable analysis, ΔWAZ was not significantly associated with antibody levels (β = −0.063, *p* = 0.571). Maternal HBsAg positivity showed no significant association (β = −0.104, *p* = 0.792). Maternal thyroid disease was independently associated with higher antibody levels (β = 0.793, 95% CI: 0.213–1.373, *p* = 0.007). None of the other covariates reached statistical significance. **Conclusions:** Hepatitis B vaccination demonstrated high immunogenicity in LBW infants, with very high seroprotection rates. Postnatal catch-up growth did not independently influence antibody levels. The significant positive association between maternal thyroid disease and infant antibody response warrants further prospective investigation.

## 1. Introduction

Globally, an estimated 296 million people are chronically infected with hepatitis B virus (HBV), leading to approximately 820,000 deaths annually from cirrhosis and hepatocellular carcinoma [1]. Universal infant hepatitis B (HepB) vaccination at birth is the cornerstone of HBV elimination [2]. However, suboptimal vaccine responses in vulnerable populations remain a concern. A 2020 cross-sectional study in Ho Chi Minh City, Vietnam, reported a seroprotection rate of only 68.3% among infants aged 12–24 months, highlighting regional challenges in achieving sustained protection [3]. Furthermore, WHO 2024 data indicate that hepatitis B vaccination coverage in the South East Asia Region averages approximately 86%, underscoring persistent regional disparities [4]. China continues to bear a substantial proportion of the global hepatitis B burden, with an estimated 70 million chronic HBV infections, making the optimization of vaccination strategies for high-risk subgroups a public health priority [5].

Low-birth-weight (LBW, <2500 g) infants represent a population of interest for vaccine-induced immunity [6]. Globally, an estimated 19.8 million babies were born with low birth weight in 2020, accounting for approximately 14.7% of all live births, or 1 in every 7 newborns [7]. In China, the low birthweight rate has shown an increasing trend, rising from 2.52% in 1992 to 3.70% in 2021, and is projected to reach 5.28% by 2030 [8]. This rising trend underscores the growing clinical and public health importance of ensuring effective vaccine responses in this population. LBW infants are especially vulnerable due to the ongoing maturation of their immune system during the neonatal period, which may affect both the magnitude and quality of vaccine responses. This vulnerability is attributed to incomplete maturation of lymphoid tissues, reduced antigen-presenting cell function, and altered cytokine production patterns during the neonatal period. Comparative studies have generally found that low birth weight alone does not significantly affect the immunogenicity of hepatitis B vaccination, with no significant difference in antibody titers or seroconversion rates when compared to normal-birth-weight infants [9,10]. For example, a comprehensive review of 15 years of safety surveillance found that seropositivity rates and antibody concentrations to hepatitis B and Hib appeared lower in infants with a history of prematurity/low birth weight than in term infants [11]. However, the potential modifying roles of postnatal catch-up growth and maternal–neonatal characteristics remain poorly understood. To date, much of the available evidence on LBW infant vaccine responses comes from single-center studies or relatively small cohorts, limiting the generalizability of findings. Large-scale, real-world data from routine immunization programs remain relatively limited in this population, particularly in China, which hampers the development of evidence-based immunization policies for LBW infants. In the context of China’s evolving hepatitis B epidemiology, which continues to show regional and demographic heterogeneity, targeted interventions for this vulnerable population are increasingly recognized as essential to achieving the nation’s elimination goals [5].

Malnutrition is one of the most important modifiable determinants of vaccine immunogenicity in early life, affecting immune function through multiple pathways [12]. Emerging evidence links nutrition to vaccine immunogenicity. A 2022 systematic review concluded that undernourished children exhibit significantly lower seroconversion rates for oral polio and rotavirus vaccines, due in part to impaired gut-barrier function and reduced lymphocyte availability [13]. Beyond gut-barrier integrity, undernutrition also impairs thymic development and lymphocyte proliferation, compromising both humoral and cell-mediated responses [12]. Early life is a critical window for immune programming: malnutrition causes thymic atrophy and lymphocytopenia, but catch-up growth can restore immune competence [14]. Importantly, this restoration is not limited to weight gain; it also involves the normalization of cytokine profiles and the recovery of lymphoid tissue function, particularly when nutritional rehabilitation occurs during the first year of life [12,14]. We therefore hypothesized that, in the absence of severe malnutrition, the degree of postnatal catch-up growth, measured as the change in weight-for-age Z-score between 6 and 8 months (ΔWAZ), would be independently associated with hepatitis B virus surface antibody anti-HBs levels in LBW infants, even after adjusting for gestational age, birth weight, and vaccine regimen.

To test this hypothesis, we conducted a retrospective cohort study of 511 LBW infants who completed the HepB vaccination series. Our primary objectives were: (i) to describe the overall anti-HBs geometric mean concentration (GMC) and seroprotection rate; (ii) to compare these outcomes across clinically relevant subgroups; and (iii) to evaluate the independent association between ΔWAZ and anti-HBs levels after adjusting for maternal characteristics (including metabolic and thyroid status), infant characteristics, and other relevant confounders.

## 2. Materials and Methods

### 2.1. Study Design and Participants

This retrospective cohort study was conducted in Taizhou City, Jiangsu Province, China, using data collected from routine immunization and maternal health surveillance systems. The study population comprised LBW infants born between 1 July 2023, and 30 June 2024, with data extraction performed in October 2025 from the two provincial information systems. Pediatric immunization is delivered through 40 hospital-based obstetric vaccination clinics (birth dose) and 141 township- and street-level clinics (subsequent doses). According to the National Immunization Program guidelines in China, the birth dose of HepB (10 μg recombinant yeast HBsAg) is given immediately after birth; hepatitis B immunoglobulin (HBIG, 100 IU anti-HBs) is provided free to infants of HBsAg-positive mothers.

Vaccination schedule for LBW infants was stratified by maternal HBsAg status and birth weight [6]. For HBsAg-negative mothers, a standard 3-dose series (0–1–6 months) was administered. For HBsAg-positive mothers, a 3-dose series (0–1–6 months) was given if birth weight ≥ 2000 g, whereas a 4-dose series (0–1–2–7 months) was used if birth weight < 2000 g. Consistent with the National Immunization Program, all infants born to HBsAg-positive mothers received hepatitis B immunoglobulin (HBIG) within 12 h of birth.

Immunization records were retrieved from the Jiangsu Provincial Vaccination Comprehensive Service Management Information System, which captures individual perinatal variables (including sex, birth date, birth weight, gestational age, Apgar score, delivery mode, and maternal HBsAg status). Maternal sociodemographic and clinical characteristics (age, BMI, blood pressure, fasting glucose, thyroid-stimulating hormone, and pregnancy complications) were obtained from the Jiangsu Maternal and Child Health Information System. Maternal comorbidities, including thyroid disease, diabetes, anemia, hypertension, scarred uterus, and a history of preterm birth, were defined based on documented medical history or clinical diagnosis recorded in the maternal health system at early pregnancy registration, rather than on a single laboratory measurement; “any maternal complication” was defined as the presence of one or more of these conditions. Maternal TSH and fasting glucose were measured during routine first-trimester screening and retrieved from the same system. All data were extracted from the two provincial information systems following standardized protocols, ensuring data completeness and consistency across the study period.

### 2.2. Sample Size Estimation

Sample size was estimated with a two-sided significance level of 0.05 and a statistical power of 80%. A priori power analysis assuming a 15% non-seroprotection rate indicated that 467 infants were required to support a logistic regression with seven prespecified covariates (maternal age, parity, gestational age, birth weight, Apgar score, delivery mode, and maternal HBsAg status). Although our cohort of 511 infants met this requirement, the observed non-seroprotection rate was extremely low (0.59%, *n* = 3). Owing to this near-ceiling effect limiting binary analysis, we focused on linear regression of log-transformed anti-HBs levels using the 462 complete cases, which provided sufficient statistical power for the specified models.

### 2.3. Participant Selection

**Figure 1:**
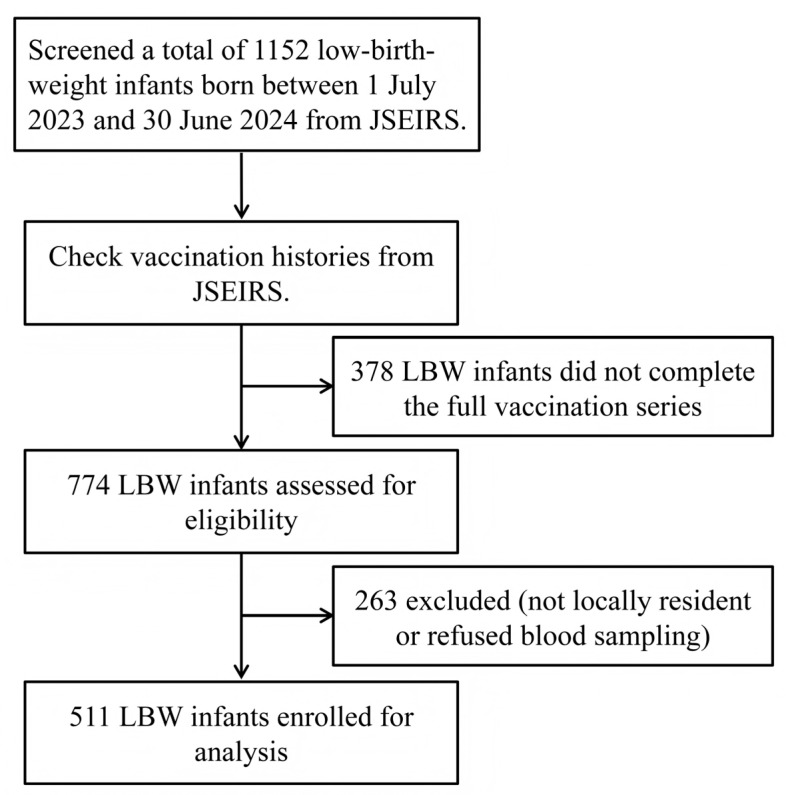
Overview of screening process.

Infants were included if they had a recorded birth weight of <2500 g, completed the recommended vaccination series, and had documented anti-HBs results measured 4–6 weeks after the final dose. Infants with incomplete vaccination records, missing key growth or laboratory variables, or relocation out of Taizhou during the study period were excluded. From 1152 eligible LBW infants initially screened, 378 were excluded because they did not complete the full vaccination series, and another 263 were excluded because they moved out of Taizhou or refused blood collection. Ultimately, 511 LBW infants were included (Figure 1). For all 22 infants born to HBsAg-positive mothers, post-vaccination HBsAg testing via the national maternal and child health surveillance system confirmed negativity.

Ethics approval was obtained from the Institutional Ethics Committee of Taizhou City Hospital of Traditional Chinese and Western Medicine (Approval No. 20223–002). Written informed consent was obtained from the parents/guardians of all participants.

### 2.4. Specimen Collection and Testing

Venous blood (2–3 mL) was collected 4–6 weeks after completion of the full hepatitis B vaccination series. Serum was separated and stored at −20 °C. Anti-HBs was quantified using a chemiluminescent immunoassay (CLIA) on a Maccura i1000 analyzer (Maccura, Chengdu, China; kit lot 1124091). The quantitative range was 0–1000 mIU/mL. Values ≥ 10 mIU/mL were considered protective (seroprotection); values < 10 mIU/mL were considered non-protective.

### 2.5. Growth Assessment

Weight and length were obtained from the Jiangsu Maternal and Child Health Information System at 1 month/42 days, 3, 6, 8, and 12 months. Weight-for-age Z-scores (WAZ) were calculated using the WHO 2006 growth standards [15] (the anthro, version 1.1.0, World Health Organization, Geneva, Switzerland). Gestational age was used as a continuous covariate in all analyses involving growth parameters to adjust for prematurity. Catch-up growth was defined as the change in WAZ from 6 to 8 months (ΔWAZ = WAZ_8m − WAZ_6m).

### 2.6. Statistical Analysis

Analyses were performed using R software, version 4.5.0 (R Foundation for Statistical Computing, Vienna, Austria). Seroprotection rate (SPR) was defined as the proportion of infants with anti-HBs ≥ 10 mIU/mL. Geometric mean concentrations (GMCs) and their 95% confidence intervals (CIs) were calculated after a natural log transformation. For statistical testing, antibody concentrations below the detection limit were assigned 5 mIU/mL before log transformation.

Group comparisons of GMCs were performed using Student’s *t*-test (two groups) or one-way ANOVA (≥3 groups), with post hoc Tukey’s test when appropriate. Comparisons of SPRs used chi-square or Fisher’s exact test (if expected cell count < 5). All tests were two-sided, and a *p*-value < 0.05 was considered statistically significant.

Univariable linear regression was performed for each candidate variable, including maternal thyroid-stimulating hormone (TSH), fasting glucose, and thyroid disease. A multivariable linear regression model was then built incorporating clinically relevant variables and those with *p* < 0.2 in univariable analysis. A separate “full model” additionally adjusted for maternal TSH, fasting glucose, thyroid disease, and maternal diabetes. Variance inflation factors (VIF) were examined to rule out multicollinearity. Model assumptions were verified by inspecting residual plots for homoscedasticity and normality, and no significant violations were detected.

Sensitivity analyses were conducted by: (i) excluding infants of HBsAg-positive mothers; (ii) replacing ΔWAZ with daily weight gain (g/day); (iii) restricting to preterm infants; and (iv) adjusting for maternal TSH and fasting glucose (as continuous or categorical variables). Figures were generated using the ggplot2 package (version 3.5.1, Wickham H, Springer-Verlag New York, 2016).

## 3. Results

### 3.1. Study Population and Overall Immune Response

After exclusions, 511 LBW infants were included (Figure 1). The mean gestational age was 34.47 weeks (SD = 2.29), the mean birth weight was 2099.32 g (SD = 338.47), and 51.27% were male. Baseline characteristics are shown in Table 1. Among these infants, 86.50% were preterm, 69.28% were delivered by cesarean section, and 19.37% were exclusively breastfed during the first 6 months. Maternal comorbidities included thyroid disease (6.85%), diabetes (7.63%), and any maternal complication (20.74%). Infant anemia at 6 months was present in 10.00% of the cohort. Sample sizes varied across maternal and infant characteristics due to missing data, with all multivariable models based on 462 infants with complete growth data.

The overall SPR (anti-HBs ≥ 10 mIU/mL) was 99.41% (508/511). The GMC was 1045.37 mIU/mL (95% CI: 916.24–1192.70), and the median was 1048.48 mIU/mL (IQR: 351.61–2811.62). Among the 22 infants born to HBsAg-positive mothers, the SPR was 95.45% (21/22).

### 3.2. Subgroup Comparisons

**Figure 2:**
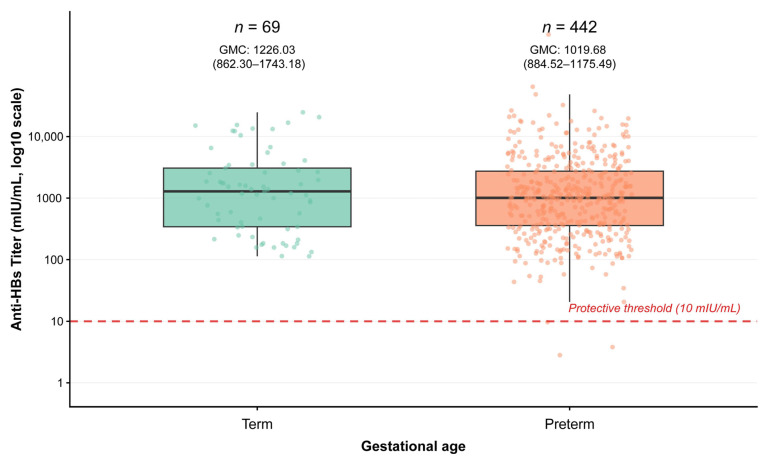
Log-transformed anti-HBs antibody levels by gestational age subgroups. Greenish-blue dots represent the Term group; coral-colored dots represent the Preterm group. Boxes show IQR, medians (horizontal lines), and whiskers (1.5 × IQR). A dashed red line indicates the protective threshold (10 mIU/mL).

**Figure 3:**
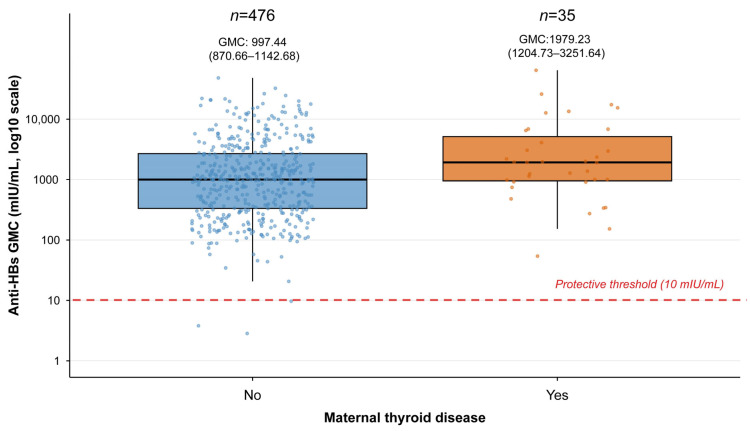
Log-transformed anti-HBs antibody levels by maternal thyroid disease status. Blue dots represent the No thyroid disease group; orange-yellow dots represent the Yes thyroid disease group. Boxes show IQR, medians (horizontal lines), and whiskers (1.5 × IQR). The dashed red line indicates the protective threshold (10 mIU/mL).

SPR were uniformly high (>99%) across all subgroups. GMC differences were not significant for gestational age, sex, delivery mode, feeding practice, maternal HBsAg status, anemia, TSH, fasting glucose, or hypertension (all *p* > 0.05). The only exception was maternal thyroid disease, where infants of affected mothers had a significantly higher GMC (1979.23 vs. 997.44 mIU/mL, *p* = 0.013). GMC differences by maternal diabetes status were not statistically significant (1051.34 mIU/mL vs. 975.70 mIU/mL, *p* = 0.768), and SPR remained high in both groups (99.36% vs. 100.00%). The distribution of log-transferred antibody levels by gestational age and the comparison between infants of mothers with and without thyroid disease are shown in Figure 2 and Figure 3, respectively. Detailed data are provided in Supplemental Table S1.

### 3.3. Catch-Up Growth and Antibody Levels

**Figure 4:**
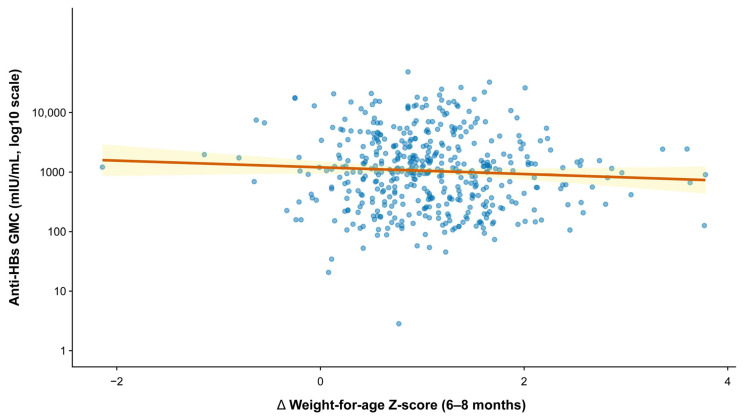
Scatter plot of the association between ΔWAZ and the log-transformed anti-HBs antibody level. Blue dots represent individual observations; the red line represents the linear regression fit, with the yellow shaded area indicating the 95% CI.

Simple linear regression showed no significant association between ΔWAZ from 6 to 8 months of age and log-transformed antibody levels (β = −0.107, *p* = 0.265; Figure 4). Multivariable linear regression adjusting for gestational age, maternal HBsAg positivity, maternal BMI, maternal fasting glucose, maternal thyroid disease, infant hemoglobin at 6 months, and ΔWAZ confirmed that ΔWAZ remained non-significant (β = −0.063, 95% CI: −0.280–0.155, *p* = 0.571). Maternal HBsAg positivity was not significantly associated with antibody levels (β = −0.104, 95% CI: −0.877–0.669, *p* = 0.792). Notably, maternal thyroid disease was independently associated with higher antibody levels (β = 0.793, 95% CI: 0.213–1.373, *p* = 0.007). The model *R*^2^ was 0.025 and the adjusted *R*^2^ was 0.005 (*p* for the overall model = 0.438). In univariate analysis, maternal age showed a significant association with log-transformed antibody levels (β = −0.035, 95% CI: −0.065 to −0.004, *p* = 0.024); however, this association did not persist in the multivariable model. Full results are given in Table 2, and univariable associations for all candidate variables are provided in Supplementary Table S2.

### 3.4. Sensitivity Analyses

Sensitivity analyses (Table 3; Supplemental Table S3) consistently showed no significant association between ΔWAZ and antibody levels. Excluding infants of HBsAg-positive mothers, using daily weight gain instead of ΔWAZ (*n* = 462), or restricting the analysis to preterm infants (*n* = 442) did not alter this null finding. Adjusting for maternal thyroid disease and maternal diabetes in the complete-case set (*n* = 462; Supplemental Table S3, Model C) also left ΔWAZ non-significant (β = 0.018, *p* = 0.889), whereas maternal thyroid disease itself remained significantly associated with higher antibody levels (β = 0.671, 95% CI: 0.031–1.312, *p* = 0.040). The model fit for this specific analysis was *R*^2^ = 0.023, adjusted *R*^2^ = 0.008. Adjusting for maternal TSH or fasting glucose (continuous or categorical) likewise did not affect the null result for ΔWAZ. Univariate analysis of maternal TSH alone also showed no association (β = −0.074, 95% CI: −0.201 to 0.053, *p =* 0.254; Supplementary Table S2).

## 4. Discussion

In this cohort of 511 LBW infants, we observed a seroprotection rate of 99.41% and a GMC of 1045.37 mIU/mL after primary hepatitis B vaccination, confirming that current protocols are highly immunogenic even in this vulnerable population [1,2,6]. This high efficacy aligns with the broader success of China’s hepatitis B immunization program [5,6,13] and contrasts sharply with reports from regions with lower vaccine coverage [3]. These findings demonstrate that LBW infants can achieve high seroprotection rates when the full vaccination series is completed on time, reinforcing the effectiveness of current immunization protocols and the importance of timely completion of all doses. From a public health perspective, this observation is particularly relevant for China, where the LBW rate has risen from 2.52% in 1992 to 3.70% in 2021 and is projected to reach 5.28% by 2030 [8], reflecting a growing population of LBW infants who may benefit from optimized immunization strategies and ensuring adequate protection in this growing vulnerable group is critical for achieving the national hepatitis B elimination goals [5]. The near-universal seroprotection achieved in this real-world cohort provides strong empirical support for the effectiveness of China’s current hepatitis B immunization strategy in LBW infants. While the overall vaccine response was excellent, we found that low birth weight alone was not a determinant of poor immunogenicity, consistent with previous meta-analyses that reported preterm birth rather than low birth weight as the key risk factor for reduced antibody responses [9,11,16]. Nevertheless, even with high final seroprotection, delayed seroconversion in LBW infants could increase the risk of perinatal transmission, and incomplete vaccination schedules may lead to poorer responses. During sample collection, we also identified LBW infants from the system who met our inclusion criteria but had not yet completed the full immunization series; active follow-up and timely reminders are warranted to ensure they complete the schedule as soon as possible.

After adjusting for gestational age, the principal finding of this study is the lack of a significant association between postnatal catch-up growth (ΔWAZ) and hepatitis B surface antibody levels. This null finding persisted across all sensitivity analyses. The consistency of this result across different analytical approaches—including alternative growth metrics and subgroup restrictions—reinforces the validity of our observation and suggests that the absence of an association is unlikely to be an artifact of a single analytical method. While malnutrition is known to impair vaccine immunogenicity, nutritional rehabilitation can enhance responses [12,13,14], our results suggest that in a cohort where severe malnutrition is uncommon (only 10.0% anemic, reflecting the overall adequate nutritional status of the study population), the velocity of postnatal growth does not determine the magnitude of the humoral response to the hepatitis B vaccine. According to WHO/UNICEF infant and young child feeding (IYCF) guidelines, breastfeeding rates are defined as measures of feeding behaviors rather than direct nutritional status indicators [17]. In our cohort, the exclusive breastfeeding rate before 6 months was 19.4%, which is considerably lower than the WHO global target of at least 50% by 2025. Recent studies have shown that breastfeeding benefits infant immune response to hepatitis B vaccine [18], while undernutrition is associated with reduced antibody responses to vaccines [19]. Although subgroup analysis in our cohort did not show a statistically significant difference in antibody levels by feeding type (Supplemental Table S1), this finding is consistent with the concept that in adequately nourished populations, additional growth velocity does not confer further humoral immune benefit, although direct evidence remains limited [20]. The absence of association between growth velocity and antibody levels in this adequately nourished cohort suggests that catch-up growth may be more relevant for other health outcomes, such as neurodevelopment or metabolic health, rather than vaccine-induced humoral immunity.

However, previous research has shown that catch-up growth in preterm infants may improve innate immune function and reduce the risk of infections [21]; thus, while antibody responses to HepB appear unaffected, broader immune benefits from catch-up growth cannot be ruled out. The mechanisms underlying this dissociation between humoral and innate immune outcomes remain unclear, but may involve distinct pathways—growth factors and metabolic hormones may preferentially influence innate immune cell development rather than antigen-specific antibody production. Moreover, a growing body of evidence suggests that the composition of the gut microbiota, which is influenced by early nutrition and growth, can modulate vaccine responses [22,23,24]; this area warrants further investigation in LBW infants. Given the unique nutritional and developmental characteristics of LBW infants, understanding the interplay between early feeding practices, gut microbiota colonization, and vaccine responses may inform targeted interventions to optimize immune outcomes in this population.

Maternal HBsAg positivity was not significantly associated with antibody levels in the primary multivariable model (β = −0.104, 95% CI: −0.877 to 0.669, *p* = 0.792), although the GMC in HBsAg-positive mothers was numerically lower (615.69 mIU/mL) than in negative mothers (1070.57 mIU/mL). This aligns with previous work showing that infants of HBsAg-positive mothers can achieve adequate seroprotection despite slightly lower antibody concentrations [25,26]. In China, the national policy provides free hepatitis B immunoglobulin (HBIG) to all infants born to HBsAg-positive mothers within 12 h of birth, followed by the standard vaccination series [25]. This integrated approach has been associated with substantial reductions in perinatal transmission rates in China [25]. A large 2023 study found that maternal HBeAg temporarily reduced anti-HBs titers at 7–12 months, but the effect was negligible by 24 months [26]. The lower GMC in this subgroup, however, suggests that long-term antibody persistence should be monitored, as reported in a 5-year follow-up study of preterm children [27].

An unexpected and novel finding was the significant positive association between maternal thyroid disease (clinical diagnosis) and infant antibody levels. In the primary model, maternal thyroid disease was independently associated with higher antibody levels (β = 0.793, 95% CI: 0.213–1.373, *p* = 0.007). This association remained robust in sensitivity analyses, including a model that additionally adjusted for maternal diabetes (β = 0.671, 95% CI: 0.031–1.312, *p* = 0.040). Autoimmune thyroiditis affects 2–17% of pregnant women, and its prevalence among women of childbearing age in China has been rising, with a national survey reporting that 14.28% have subclinical hypothyroidism and 13.53% are positive for thyroid peroxidase antibodies [28]. It is well established that maternal anti-thyroid antibodies can cross the placenta and may cause transient thyroid dysfunction in newborns [29,30,31]. A recent prospective study found that 94% of newborns of mothers with autoimmune thyroiditis had detectable anti-thyroid antibodies at first evaluation [29]. These maternal IgG antibodies may act as non-specific adjuvants, enhancing the infant’s B-cell response to vaccination. This adjuvant-like effect may be mediated through Fc receptor engagement on B cells or through the formation of immune complexes that enhance antigen presentation and germinal center reactions, thereby facilitating more robust antibody production. Maternal thyroid autoimmunity has been shown to affect natural killer cell generation and inflammatory cytokine secretion in cord blood, and maternal antibodies can shape the neonatal B-cell repertoire [32,33]. To our knowledge, this is the first study to report such an association in LBW infants. Importantly, neither continuous nor categorical maternal TSH levels were associated with infant antibody levels (univariate *p* = 0.254; multivariate *p* > 0.30), suggesting that the observed effect is attributable to thyroid autoantibodies rather than to thyroid hormone dysregulation per se. A large meta-analysis of factors affecting long-term protection after infant hepatitis B vaccination also found no significant association between maternal TSH levels and antibody persistence [34]. Consistent with this, anti-thyroid antibodies can undergo changes in maternal autoimmune thyroiditis, resulting in different clinical effects in different pregnancies. This exploratory finding warrants further investigation in larger and more diverse populations, including term and near-term infants. Given the rising prevalence of thyroid disorders among Chinese women of childbearing age, this finding has important implications for prenatal care and neonatal vaccination strategies. If confirmed in future studies, this association could have implications for prenatal risk stratification and personalized immunization approaches, although the relatively small number of affected mothers (*n* = 35) in our cohort necessitates further validation in larger studies.

Several considerations warrant cautious interpretation. The relatively low *R*^2^ value (0.025) observed in the multivariable model is not unexpected in vaccine immunogenicity studies, as antibody responses are influenced by a broad range of genetic, maternal, and environmental factors beyond those captured in our analysis. The retrospective design inherently precludes causal inference, and residual confounding may persist despite multivariable adjustment. Discrete growth measurement timepoints may not fully capture the dynamic trajectory of catch-up growth, and our primary multivariable models were based on 462 infants with complete growth data, which may introduce selection bias if the excluded infants differed systematically from those included. Additionally, missing data on maternal TSH and fasting glucose reduced the sample size for extended models from 511 to 293, reflecting the real-world nature of retrospective data extraction from routine health information systems. This reduction may have diminished statistical power to detect small effects. Additionally, the high seroprotection rate (99.41%) limited our ability to identify predictors of seroconversion failure, and the single-center design in one Chinese prefecture may restrict generalizability to other populations or healthcare settings.

## 5. Conclusions

Hepatitis B vaccination induces effective immune responses in LBW infants, with near-universal seroprotection. After adjusting for gestational age, postnatal catch-up growth was not identified as an independent determinant of antibody levels, reinforcing the importance of timely completion of the full vaccination series regardless of variations in growth patterns. These findings suggest that growth velocity may not be the primary concern for vaccine efficacy in adequately nourished LBW infants. The exploratory finding that maternal thyroid disease is associated with higher infant antibody levels warrants prospective confirmation with direct measurement of thyroid autoantibodies. This study provides contemporary, real-world evidence from a large Chinese LBW cohort, addressing a critical gap in the literature regarding the role of postnatal growth velocity and maternal thyroid status in modulating vaccine responses. From a public health perspective, these findings support the continued implementation of current hepatitis B immunization strategies for LBW infants and highlight the importance of post-vaccination serological monitoring in this population to ensure sustained protection. Clinicians should continue to follow existing vaccination guidelines for LBW infants, which have proven remarkably successful.

## Figures and Tables

**Table 1:** Baseline characteristics of the 511 low-birth-weight infants.

C.	N	Value
Gestational age (weeks), mean (SD)	511	34.47 ± 2.29
Birth weight (g), mean (SD)	511	2099.32 ± 338.47
Preterm (<37 weeks), *n* (%)	511	442 (86.50%)
Male, *n* (%)	511	262 (51.27%)
Cesarean section, *n* (%)	511	354 (69.28%)
Exclusive breastfeeding (first 6 months), *n* (%)	511	99 (19.37%)
Maternal HBsAg positive, *n* (%)	511	22 (4.31%)
Maternal BMI (kg/m^2^), mean (SD)	506	22.99 ± 4.04
Maternal fasting glucose (mmol/L), mean (SD)	415	4.86 ± 1.00
Maternal TSH (mIU/L), mean (SD)	293	1.92 ± 1.44
Maternal thyroid disease, *n* (%)	511	35 (6.85%)
Maternal diabetes, *n* (%)	511	39 (7.63%)
Infant hemoglobin at 6 months (g/L), mean (SD)	450	118.17 ± 10.75
Anemia at 6 months (Hb < 110 g/L), *n* (%)	450	45 (10.00%)
Catch-up growth (ΔWAZ 6–8 months), mean (SD)	462	1.08 ± 0.72
Any maternal complication, *n* (%)	511	106 (20.74%)

Note: SD, standard deviation. Preterm refers to the current pregnancy. Sample sizes vary due to missing data: maternal BMI (*n* = 506), infant hemoglobin at 6 months (*n* = 450), maternal fasting glucose (*n* = 415), and maternal TSH (*n* = 293). All multivariable models were based on complete cases (*n* = 462).

**Table 2:** Multivariable linear regression for log-transformed anti-HBs levels (mIU/mL).

Variable	β (95% CI)	*p* Value
Gestational age (per week)	0.017 (−0.053, 0.086)	0.639
Maternal HBsAg (positive vs. negative)	−0.104 (−0.877, 0.669)	0.792
Maternal BMI (per kg/m^2^)	−0.006 (−0.043, 0.031)	0.761
Maternal fasting glucose (per mmol/L)	−0.035 (−0.183, 0.114)	0.648
Maternal thyroid disease (yes vs. no)	0.793 (0.213, 1.373)	0.007
Infant hemoglobin at 6 months (per g/L)	−0.002 (−0.018, 0.013)	0.775
ΔWAZ (per 1 unit)	−0.063 (−0.280, 0.155)	0.571

Note: CI, confidence interval; ΔWAZ = change in weight-for-age Z-score from 6 to 8 months. Model fit: *R*^2^ = 0.025, adjusted *R*^2^ = 0.005, *p* for model = 0.438.

**Table 3:** Key sensitivity analyses (summary).

Sensitivity Analysis	Variable	β (95% CI)	*p* Value
Excluding HBsAg-positive mothers	ΔWAZ	−0.120 (−0.311, 0.070)	0.214
Using daily weight gain (g/day)	Weight gain	−0.010 (−0.028, 0.008)	0.274
Restricting to preterm infants	ΔWAZ	−0.154 (−0.353, 0.045)	0.129
Adjusting for maternal thyroid disease	ΔWAZ	0.018 (−0.234, 0.270)	0.889
Adjusting for maternal thyroid disease	Thyroid disease (yes vs. no)	0.671 (0.031, 1.312)	0.040 *

Note: The sample sizes were 489 for excluding HBsAg-positive mothers, 462 for using daily weight gain, and 442 for restricting to preterm infants. The model adjusting for maternal thyroid disease also included maternal diabetes as a covariate and used the same complete-case set as the primary model (*n* = 462). For its model fit, see Supplemental Table S3, Model C (*R*^2^ = 0.023, adjusted *R*^2^ = 0.008). * *p* < 0.05.

## Data Availability

The raw data supporting the conclusions of this article will be made available by the corresponding author upon reasonable request. The authors declare that no Generative AI was used in the creation of this manuscript.

## Supplementary Materials

The following supporting information can be downloaded at: https://www.mdpi.com/article/10.3390/vaccines14070566/s1, Table S1: Geometric mean concentration (GMC) and seroprotection rate (SPR) by subgroups; Table S2: Univariate linear regression analysis for log-transferred anti-HBs levels (mIU/mL); Table S3: Extended sensitivity analyses: adjusting for maternal TSH, glucose, thyroid disease, and GDM (complete case, *n* = 293).
