# Supplementary material for: Evaluation of Hepatitis B Vaccine Immunogenicity in Low-Birth-Weight Infants After Complete Immunization: The Impact of Postnatal Catch-Up Growth and Maternal–Neonatal Characteristics
Source: Vaccines (Basel). 2026 Jun 27;14(7):566. doi: 10.3390/vaccines14070566 (PMC13417890; doi:10.3390/vaccines14070566)
Supplement: Supplementary file 1 [file vaccines-14-00566-s001.zip › vaccines-4369349-supplementary.pdf]

Supplemental Table S1 Geometric mean concentration (GMC) and seroprotection rate (SPR) by subgroups

| **Subgroup** | **Level** | **N** | **GMC (mIU/mL) (95% CI)** | **SPR (%)** |
|---|---|---|---|---|
| Gestational age | Preterm | 442 | 1019.68 (884.52–1175.49) | 99.32 |
| | Term | 69 | 1226.03 (862.30–1743.18) | 100.00 |
| Sex | Female | 249 | 1007.67 (834.62–1216.60) | 99.20 |
| | Male | 262 | 1082.51 (899.85–1302.24) | 99.62 |
| Delivery mode | Cesarean | 354 | 1065.71 (906.58–1252.77) | 99.15 |
| | Vaginal | 157 | 1000.92 (797.75–1255.83) | 100.00 |
| Feeding | Breastfeeding | 99 | 1005.26 (752.30–1343.27) | 100.00 |
| | Formula | 115 | 989.43 (754.18–1298.05) | 99.13 |
| | Mixed | 297 | 1081.89 (906.63–1291.03) | 99.33 |
| Maternal HBsAg | Negative | 489 | 1070.57 (936.38–1223.99) | 99.59 |
| | Positive | 22 | 615.69 (306.15–1238.20) | 95.45 |
| Anemia at 6 months | No anemia | 405 | 1000.54 (864.23–1158.36) | 99.51 |
| | Anemia | 45 | 1335.40 (908.99–1961.85) | 100.00 |
| Maternal TSH > 2.5 mIU/L | Normal | 214 | 1189.92 (967.28–1463.80) | 99.53 |
| | High | 79 | 986.13 (674.16–1442.48) | 97.47 |

| Subgroup | Level | N | GMC (mIU/mL) (95% CI) | SPR (%) |
|---|---|---|---|---|
| Maternal fasting glucose ≥ 5.6 mmol/L | Normal | 364 | 1063.10 (911.10–1240.47) | 99.45 |
| | High | 51 | 1062.89 (699.03–1616.16) | 100.00 |
| Maternal hypertension | Normotensive | 490 | 1030.43 (900.67–1178.90) | 99.39 |
| | Hypertensive | 18 | 1469.95 (723.36–2987.08) | 100.00 |
| Maternal thyroid disease | No | 476 | 997.44 (870.66–1142.68) | 99.37 |
| | Yes | 35 | 1979.23 (1204.73–3251.64) | 100.00 |
| Maternal diabetes | No | 472 | 1051.34 (916.70–1205.76) | 99.36 |
| | Yes | 39 | 975.70 (598.30–1591.15) | 100.00 |

Note: TSH high was defined as >2.5 mIU/L; fasting glucose high was defined as ⩾5.6 mmol/L.

Supplementary Table S2. Univariate linear regression analysis for log-transferred anti-HBs levels (mIU/mL)

| **Variable** | **Type** | **Level** | **N** | **Estimate (β)** | **95% CI** | ***p* value** |
|---|---|---|---|---|---|---|
| Gestational age | Continuous | – | 511 | 0.049 | –0.009, 0.106 | 0.098 |
| Birth weight | Continuous | – | 511 | 0.000 | 0.000, 0.000 | 0.938 |
| Mother age | Continuous | – | 511 | - 0.035 | –0.065, –0.004 | 0.024 |
| Father age | Continuous | – | 508 | - 0.025 | –0.055, 0.006 | 0.112 |
| Mother BMI | Continuous | – | 506 | - 0.003 | - 0.036, 0.030 | 0.874 |
| Mother SBP | Continuous | – | 508 | 0.006 | - 0.005, 0.018 | 0.272 |
| Mother DBP | Continuous | – | 508 | 0.003 | - 0.012, 0.018 | 0.672 |
| Mother fasting glucose | Continuous | – | 415 | - 0.034 | - 0.178, 0.111 | 0.648 |
| Mother TSH | Continuous | – | 293 | - 0.074 | - 0.201, 0.053 | 0.254 |
| Δ WAZ | Continuous | – | 462 | - 0.121 | - 0.310, 0.069 | 0.211 |
| Daily weight gain | Continuous | – | 462 | - 0.011 | - 0.029, 0.007 | 0.221 |
| Sex | Categorical | Male | 262 | 0.072 | - 0.193, 0.336 | 0.595 |
| Delivery mode | Categorical | Vaginal | 157 | - 0.063 | - 0.349, 0.224 | 0.666 |
| Feeding | Categorical | Formula | 115 | - 0.016 | - 0.426, 0.394 | 0.833 |
| Feeding | Categorical | Mixed | 297 | 0.074 | - 0.274, 0.421 | 0.677 |

| **Variable** | **Type** | **Level** | **N** | **Estimate (β)** | **95% CI** | ***p* value** |
|---|---|---|---|---|---|---|
| Preterm | Categorical | Term | 69 | 0.184 | − 0.202, 0.571 | 0.350 |
| Maternal HBsAg | Categorical | Positive | 22 | − 0.552 | − 1.201, 0.098 | 0.096 |
| Anemia | Categorical | No anemia | 405 | − 0.288 | − 0.747, 0.170 | 0.217 |
| TSH high | Categorical | Normal | 214 | 0.187 | − 0.226, 0.601 | 0.373 |
| FBG high | Categorical | Normal | 364 | 0.000 | − 0.442, 0.442 | 0.999 |
| Hypertension | Categorical | Normotensive | 490 | − 0.355 | − 1.072, 0.362 | 0.331 |
| Diabetes | Categorical | Yes | 39 | − 0.075 | − 0.573, 0.423 | 0.768 |
| Pregnancy hypertension | Categorical | Yes | 3 | 0.261 | − 0.578, 1.101 | 0.541 |
| Pregnancy anemia | Categorical | Yes | 42 | − 0.192 | − 0.673, 0.289 | 0.433 |
| Maternal thyroid disease | Categorical | Yes | 35 | 0.685 | 0.165, 1.205 | 0.010 |
| Any complication | Categorical | Yes | 106 | 0.207 | − 0.119, 0.532 | 0.213 |

Note: TSH high was defined as >2.5 mIU/L; FBG high was defined as ≥5.6 mmol/L. Significant *p* values ($p < 0.05$) are shown in bold. CI, confidence interval.

Supplemental Table S3 Extended sensitivity analyses: adjusting for maternal TSH, glucose, thyroid disease, and GDM (complete case, n=293)

| Model | Variable | β (95% *CI*) | *P* value |
|---|---|---|---|
| Model A (continuous TSH + glucose) | ΔWAZ | 0.024 (−0.233, 0.281) | 0.854 |
| | Maternal TSH | −0.020 (−0.147, 0.108) | 0.762 |
| | Maternal fasting glucose | −0.064 (−0.262, 0.134) | 0.524 |
| Model B (categorical TSH + glucose) | ΔWAZ | 0.024 (−0.231, 0.279) | 0.852 |
| | TSH high (vs. normal) | −0.054 (−0.476, 0.367) | 0.800 |
| | FBG elevated (vs. normal) | 0.047 (−0.556, 0.650) | 0.878 |
| Model C (thyroid disease +diabetes) | ΔWAZ | 0.018 (−0.234, 0.270) | 0.889 |
| | Maternal thyroid disease (yes vs. no) | 0.671 (0.031, 1.312) | 0.040 |
| | Maternal diabetes (yes vs. no) | 0.076 (−0.583, 0.736) | 0.820 |

Note: All models also adjusted for gestational age, maternal HBsAg, sex, and birth weight. CI, confidence interval.

Model fits: Model A: $R^2$ = 0.025, adjusted $R^2$ = −0.004; Model B: $R^2$ = 0.023, adjusted $R^2$ = −0.006; Model C: $R^2$ = 0.023, adjusted $R^2$ = 0.008.
